# Association of *ABCB1 *genetic variants with renal function in Africans and in Caucasians

**DOI:** 10.1186/1755-8794-1-21

**Published:** 2008-06-02

**Authors:** Murielle Bochud, Chin B Eap, Marc Maillard, Toby Johnson, Peter Vollenweider, Pascal Bovet, Robert C Elston, Sven Bergmann, Jacques S Beckmann, Dawn M Waterworth, Vincent Mooser, Anne Gabriel, Michel Burnier

**Affiliations:** 1 University Institute of Social and Preventive Medicine (IUMSP), Centre Hospitalier Universitaire Vaudois and University of Lausanne, Bugnon 17, Lausanne, Switzerland; 2 Unit of Biochemistry and Clinical Psychopharmacology, Center for Psychiatric Neurosciences, Department of Psychiatry, Centre Hospitalier Universitaire Vaudois and University of Lausanne Lausanne, Switzerland; 3 Division of Nephrology, Centre Hospitalier Universitaire Vaudois, Lausanne, Switzerland; 4 Department of Medical Genetics, University of Lausanne, Lausanne, Switzerland; 5 Swiss Institute of Bioinformatics, Lausanne, Switzerland; 6 Department of Medicine, Centre Hospitalier Universitaire Vaudois, Lausanne, Switzerland; 7 Ministry of Health, Victoria, Seychelles; 8 Department of Epidemiology and Biostatistics, Case Western Reserve University, Cleveland (OH), USA; 9 Service of Medical Genetics, Centre Hospitalier Universitaire Vaudois, Lausanne, Switzerland; 10 Division of Genetics, GlaxoSmithKline, Philadelphia, Pennsylvania, USA

## Abstract

**Background:**

The P-glycoprotein, encoded by the *ABCB1 *gene, is expressed in human endothelial and mesangial cells, which contribute to control renal plasma flow and glomerular filtration rate. We investigated the association of *ABCB1 *variants with renal function in African and Caucasian subjects.

**Methods:**

In Africans (290 subjects from 62 pedigrees), we genotyped the *2677G>T *and *3435 C>T ABCB1 *polymorphisms. Glomerular filtration rate (GFR) was measured using inulin clearance and effective renal plasma flow (ERPF) using para-aminohippurate clearance. In Caucasians (5382 unrelated subjects), we analyzed 30 SNPs located within and around *ABCB1*, using data from the Affymetrix 500 K chip. GFR was estimated using the simplified Modification of the Diet in Renal Disease (MDRD) and Cockcroft-Gault equations.

**Results:**

In Africans, compared to the reference genotype (GG or CC), each copy of the *2677T *and *3435T *allele was associated, respectively, with: GFR higher by 10.6 ± 2.9 (*P *< 0.001) and 4.4 ± 2.3 (*P *= 0.06) mL/min; ERPF higher by 47.5 ± 11.6 (*P *< 0.001) and 28.1 ± 10.5 (*P *= 0.007) mL/min; and renal resistances lower by 0.016 ± 0.004 (*P *< 0.001) and 0.011 ± 0.004 (*P *= 0.004) mm Hg/mL/min. In Caucasians, we identified 3 polymorphisms in the *ABCB1 *gene that were strongly associated with all estimates of GFR (smallest P value = 0.0006, overall P = 0.014 after multiple testing correction).

**Conclusion:**

Variants of the *ABCB1 *gene were associated with renal function in both Africans and Caucasians and may therefore confer susceptibility to nephropathy in humans. If confirmed in other studies, these results point toward a new candidate gene for nephropathy in humans.

## Background

A better knowledge of the determinants of renal function is of paramount importance considering the high and increasing burden of chronic kidney disease worldwide. [1] The familial aggregation of renal function [2-4] suggests that genetic factors determine, in part, renal function and that there are candidate genes for nephropathy in humans.

The transmembrane efflux-P-glycoprotein (PGP; also known as multidrug resistance-associated protein 1) is encoded by the *ABCB1 *gene, several genetic variants of which have been shown to influence PGP expression in humans. PGP has been extensively studied in a pharmacogenetic context, in particular for its role in multi-drug resistance in cancer treatment. PGP is widely expressed in the human kidney [5-7] and is involved in ciclosporin-induced post-transplantation nephrotoxicity [5,8,9], possibly because of its influence on ciclosporin absorption. [10]*ABCB1 *genetic variants have been associated with post-transplantation ciclosporin-induced nephrotoxicity. [10-12] In contrast to the extensive data about the physiological role of PGP on the transport of xenobiotics, little is known about the role of PGP in the transport of endogenous substrates and to our knowledge, *ABCB1 *gene variants have not been previously associated with renal function in the absence of xenobiotics in humans. [13]

Since PGP is expressed in human endothelial and mesangial cells, which contribute to the control of renal plasma flow and glomerular filtration, we hypothesized that variants in the *ABCB1 *gene could be associated with renal hemodynamics and glomerular filtration rate (GFR), even in the absence of treatment by PGP substrates and/or modulators, such as ciclosporin. In particular, we investigated whether the *3435 C>T *and the *2677G>T ABCB1 *variants are associated with GFR, effective renal plasma flow (ERPF) and renal vascular resistance (RVR) in families of African descent. We then confirmed an association of variants in the *ABCB1 *gene with renal function using the 30 genetic markers located within the *ABCB1 *gene in a large ongoing population-based genome-wide association study of Caucasians employing the Affymetrix 500 K chip.

## Methods

### Seychelles study

#### Study population

In the Seychelles islands (Indian Ocean, African region), we enrolled 494 subjects of East African descent from 76 families enriched in hypertensive individuals between August 1999 and January 2002. The detailed family selection process has been previously described. [2] Renal hemodynamics and *ABCB1 *genotype were determined in 297 individuals. We excluded seven individuals with extreme outlier values for any of the renal measures, i.e. observations lying beyond three interquartile ranges from the first and third quartiles, leaving 290 individuals to analyze the associations of interest. The study was approved by the Ethical Committees of the Ministry of Health in the Seychelles and of the University of Lausanne (Switzerland). All participants provided written informed consent.

#### Phenotype and covariate measurements

In the Seychelles study, antihypertensive therapy, if any, was stopped for 2 weeks before clearance protocols. After a two-hour equilibration period, two one-hour inulin and PAH clearances were obtained to measure GFR and ERPF, respectively [see Additional file 1]. The inulin and PAH clearances (C_x_) were calculated with the formula C_x _= U_x_*V/P_x_, where U_x _and P_x _are urinary and plasma concentrations of the x solute, and V is the urine flow rate in ml/min. Renal blood flow (RBF) was calculated as ERPF/(1-(haematocrit/100)) and RVR as (mean arterial blood pressure)/RBF. GFR was also estimated using the abbreviated Modification of the Diet in Renal Disease (MDRD) equation [14]. Creatinine concentration was measured by the picric acid method (Cobas-Mira, Roche, Basel, Switzerland). Mean arterial blood pressure (MAP) was calculated as diastolic blood pressure plus 1/3 of (systolic minus diastolic blood pressure) from the mean of 6 measurements taken with a mercury sphygmomanometer (3 on the day preceding clearances and 3 on the morning of the clearances). On the day preceding clearance, participants collected urine for 24 hours. Urinary and plasma sodium and potassium concentrations were measured by flame photometry (IL-943, Instrumentation Laboratory, Milan, Italy). Participants on antidiabetic treatment during the preceding month, or with fasting blood glucose ≥ 7.0 mmol/l (measured on venous whole blood in duplicate using a Glycotronic^® ^C reflectometer, Macherey-Nagel, Düren, Germany), were considered as diabetics. Body surface area was calculated using the Dubois formula. [15] Body mass index (BMI) was calculated as weight (kg) divided by squared height (m^2^).

#### Genetic analyses

DNA was isolated using standard methods from blood drawn into K-EDTA tubes and stored at 4°C. The *ABCB1 *genotypes (exon 21, *2677 G>T *[rs2032582] and exon 26, *3435 C>T *[rs1045642]) were determined by real-time PCR with TaqMan^®^, as previously described. [16,17] The marker genotypes did not significantly deviate from Hardy-Weinberg proportions (P > 0.09) in founders, and *2677 G>T *and *3435 C>T *were in strong linkage disequilibrium (D' = 0.90) with each other.

### Statistical analyses

We used the ASSOC program in S.A.G.E. (V5.3) [18], which accounts for familial correlations by implementing simultaneous maximum likelihood estimation of both familial components of variance and covariate coefficients, to conduct linear regressions with GFR, ERPF or RVR as dependent variables. Based on the findings from descriptive analyses in our sample (i.e. GT heterozygotes had phenotypic values lying between those of the GG and TT homozygotes), we assumed an additive mode of action for the *ABCB1 3435T *and *2677T *alleles. To conduct analyses stratified by hypertension status, we defined hypertensive subjects as those on antihypertensive treatment at entry into the study or having, respectively, a systolic and/or diastolic blood pressure = 140/90 mm Hg. For multiple linear regression models, we used as predictors age, sex, BMI, 24-hour urinary sodium and potassium excretion, fasting blood glucose, diabetes and MAP. All multivariable models included ascertainment as previously described. [19] We conducted sensitivity analyses that included extreme outlier values. We formally tested whether normotensive and hypertensive subjects had similar results by including a test of hypertension status by genotype interaction (i.e. a likelihood ratio test with 2 degrees of freedom) in multivariable models. To ensure that any associations were not due to population stratification: (1) because some subjects were of mixed descent, we conducted a sensitivity analysis that included only those subjects (78%) with known grand-parent's ethnicity who reported having at least 3 grand-parents of African descent (n = 153), and (2) we extended our analysis of GFR by a separate analysis of 119 subjects from the same families with missing inulin clearance but available MDRD. We also conducted analyses based on haplotypes. For the 61 doubly heterozygous participants (CT/GT) with a potentially ambiguous phase, *ABCB1 *haplotypes were inferred using the HAPLORE program that uses an expectation-maximisation (EM) algorithm in pedigree data. [20]

### CoLaus study

#### Study population

A simple non-stratified random sample (n = 56,694) representing 35% of the overall population of the city of Lausanne (Switzerland) aged 35–75 years was drawn. Inclusion criteria were: a) written informed consent; b) aged 35–75 years; c) available examination and blood sample and d) Caucasian descent. The study was approved by the ethical committee of the Faculty of Medicine of Lausanne. Recruitment began in June 2003 and ended in May 2006. Among those eligible, the participation rate was 41%. Of the 6188 participants, 781 were excluded because of missing or low quality genotyping data, genetic relatedness with other subjects, or missing phenotyping data, leaving 5407 subjects for the present analyses.

#### Phenotype and covariate measurements

Venous blood samples (50 mL) were drawn after an overnight fast, and clinical chemistry assays were performed by the CHUV Clinical Laboratory on fresh blood samples on a Modular P apparatus (Roche Diagnostics, Switzerland) within 2 hours of blood collection. Serum creatinine was measured by the Jaffe kinetic compensated method (2.9% – 0.7% maximum inter and intra-batch CVs). GFR was estimated using the abbreviated MDRD equation [14] and the Cockcroft-Gault (CG) formula [21]. Body weight and height were measured with participants standing without shoes in light indoor clothes. Smoking was defined as present if a participant reported to be a current smoker at the time of examination, and alcohol consumption as present for participants reporting drinking alcohol at least once a day. Diabetes was defined as being on current antidiabetic treatment or having fasting plasma blood glucose ≥ 7 mmol/L.

#### Genetic analyses

Nuclear DNA was extracted from whole blood for whole genome scan analysis. Genotyping was performed on 6014 participant samples, using the Affimetrix 500 K SNP chip, as recommended by the manufacturer. Individuals with less than 95% genotyping efficiency overall (or <90% efficiency on either array, N = 399), and individuals with possible gender inconsistencies (N = 5), were removed. Duplicate samples, and first and second degree relatives were identified by estimating genomic identity-by-descent (i.b.d.) coefficients; the younger individual from each such pair was removed (N = 200). A further 3 individuals had missing phenotype data, leaving 5407 individuals with complete data available for analysis. Monomorphic SNPs (N = 4052), SNPs with less than 70% genotyping efficiency (N = 157), and SNPs not in Hardy-Weinberg proportions (N = 35417) were excluded from analysis.

### Statistical analyses

A more detailed description of the statistical analyses can be viewed in the Additional file 1. We excluded individuals with outlier phenotypic values defined as values lying more than 4 SD away from the mean (N = 25), leaving 5382 individuals for association tests. Phenotype data were transformed and adjusted for covariates using R. [22] We used age, height, weight and BMI as continuous covariates, and sex, alcohol consumption, smoking, diabetes, antidiabetic or antihypertensive treatment, the presence (or absence) of each of the major classes of antihypertensive drugs (ACE-inhibitors, angiotensin receptor blockers, beta-blockers, channel calcium blockers and diuretics) and currently (i.e. during the last 6 months) taking any prescription drug as binary covariates in normal linear regression. To confirm the association, we used only directly genotyped SNPs first and imputed SNPs in further exploratory analyses. Association tests were performed using PLINK (v0.99s) [23], with an additive mode of action.

## Results

### Seychelles Study

The allele frequency was 34.1% overall and 30.8% in founders for *2677T*, and 54.5% and 53.9%, respectively, for *3435T*. Participants' characteristics by genotypes and allele carrier status are presented in Table 1. There was no significant trend across genotypes. Carriers of the *3435T *allele had a higher BMI than non-carriers (28.8 ± 0.4 versus 27.3 ± 0.5 kg/m^2^; *P *= 0.02).

**Table 1:** Distribution of selected characteristics by genotypes for each *ABCB1 *variant in the Seychelles

	***2677 G>T***			***3435 C>T***		
	**GG**	**GT**	**TT**	**CC**	**CT**	**TT**

N	191	84	15	132	118	40

Age (years)	46.8 ± 12.2	46.2 ± 11.8	44.6 ± 12.5	46.0 ± 12.2	47.3 ± 11.5	45.8 ± 13.3
Proportion of women	0.57 ± 0.50	0.51 ± 0.50	0.47 ± 0.52	0.59 ± 0.49	0.51 ± 0.50	0.53 ± 0.51
BMI^a ^(kg/m^2^)	28.0 ± 5.6	28.5 ± 5.0	26.8 ± 4.2	27.3 ± 5.2	29.0 ± 5.7	28.0 ± 4.9
MAP^b ^(mm Hg)	101 ± 13	103 ± 14	98 ± 15	100 ± 14	103 ± 13	99 ± 14
FBG^c ^(mmol/L)	4.6 ± 1.8	4.6 ± 1.7	4.5 ± 1.6	4.6 ± 1.6	4.7 ± 1.9	4.6 ± 1.5
Diabetes (prevalence)	0.08 ± 0.28	0.11 ± 0.31	0.07 ± 0.26	0.08 ± 0.27	0.09 ± 0.29	0.13 ± 0.33
Plasma Na (mmol/L)	140 ± 4	140 ± 4	139 ± 3	140 ± 4	140 ± 4	139 ± 3
Plasma K (mmol/L)	3.8 ± 0.3	3.7 ± 0.3	3.7 ± 0.4	3.7 ± 0.3	3.8 ± 0.3	3.7 ± 0.3
Urine Na (mmol/24 h)	106 ± 54	107 ± 56	104 ± 67	101 ± 54	108 ± 56	118 ± 57
Urine K (mmol/24 h)	44 ± 18	46 ± 18	35 ± 16	44 ± 17	45 ± 19	44 ± 17

Results presented are the mean ± s.d. For each variant, there was no significant trend across genotypes. ^a^BMI: body mass index. ^b^MAP: mean arterial pressure. ^c^FBG: fasting blood glucose.

**Figure 1:**
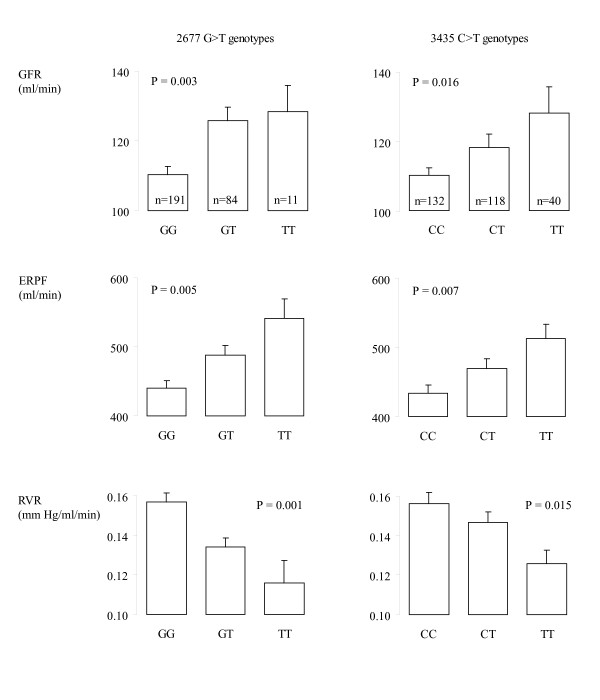
**Renal haemodynamics by genotypes for each *ABCB1 *variant**. Bar heights are means and vertical lines standard errors. P: P value for linear trend across genotypes using the ASSOC program in S.A.G.E. in models without additional covariates. GFR: glomerular filtration rate measured using inulin clearance. ERPF: effective renal plasma flow measured using PAH clearance. RVR: renal vascular resistance.

The *2677T *allele was associated, in an additive manner, with higher GFR (P = 0.003) and ERPF (P = 0.005) and lower RVR (P = 0.001) (Figure 1). Results were similar, although slightly less significant with the *3435T *allele. These results suggest that the T allele is a protective allele with respect to renal function. We obtained similar results for renal haemodynamic phenotypes adjusted for body surface area (Table 2). It is therefore unlikely that the difference in BMI observed between *3435T *carriers and non-carriers represents a major confounding factor for these associations.

**Table 2:** Renal haemodynamics by genotypes for each *ABCB1 *variant in the Seychelles

	***2677 G>T***			***3435 C>T***		
	**GG**	**GT**	**TT**	**CC**	**CT**	**TT**

N	191	84	15	132	118	40

GFR^a ^(mL/min)	110 ± 32	126 ± 35	128 ± 29^b^	110 ± 29	118 ± 37	128 ± 31^c^
GFR^a ^(mL/min/1.73 m^2^)	103 ± 25	114 ± 26	118 ± 26^b^	104 ± 24	107 ± 27	117 ± 25^c^
ERPF^d ^(mL/min)	440 ± 143	488 ± 126	542 ± 109^b^	434 ± 128	470 ± 150	513 ± 129^b^
ERPF^d ^(mL/min/1.73 m^2^)	412 ± 119	445 ± 100	500 ± 102^b^	413 ± 112	426 ± 117	470 ± 108^c^
RVR^e ^(mm Hg/min/mL)	0.16 ± 0.06	0.13 ± 0.04	0.12 ± 0.04^b^	0.16 ± 0.06	0.15 ± 0.06	0.13 ± 0.04^c^
RVR^e ^(mm Hg/min/mL/1.73 m^2^)	0.15 ± 0.07	0.13 ± 0.04	0.11 ± 0.05^b^	0.15 ± 0.07	0.14 ± 0.06	0.12 ± 0.04^c^

Results are the unadjusted mean ± s.d. ^a^GFR: glomerular filtration rate (inulin clearance). ^b ^*P *< 0.01, ^c ^0.01 <*P *< 0.05, for linear trend across genotypes coded as 0 (GG or CC), 1 (GT or CT) and 2 (TT). ^d^ERPF: effective renal plasma flow (PAH clearance). ^e^RVR: renal vascular resistance.

**Figure 2:**
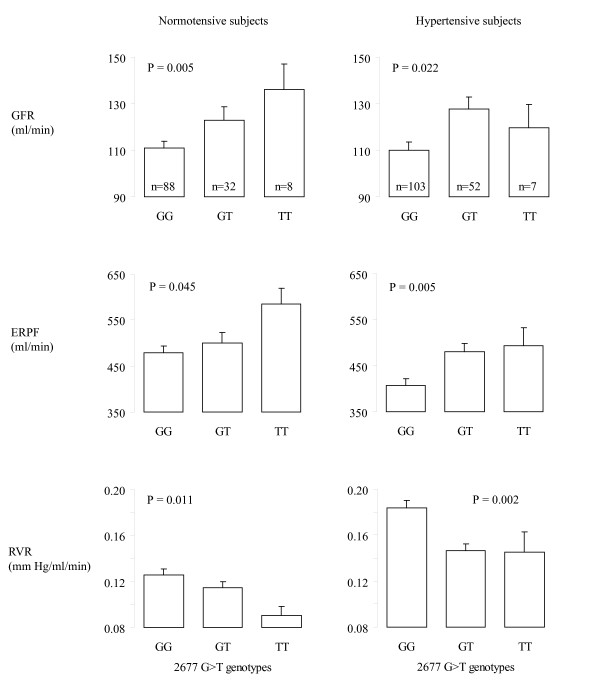
**Renal haemodynamics by hypertension status for the *2677 C>T ABCB1 *variants**. Bar heights are means and vertical lines standard errors. P: P value for linear trend across genotypes using the ASSOC program in S.A.G.E. in models without additional covariates. GFR: glomerular filtration rate measured using inulin clearance. ERPF: effective renal plasma flow measured using PAH clearance. RVR: renal vascular resistance.

Because PGP is responsible for the transport of many drugs, we conducted analyses stratified by hypertension status (Figure 2). As the association was similar in normotensive subjects and hypertensive subjects, it is unlikely that our results reflect a confounding effect of antihypertensive drugs. The hypertension status by genotype interaction was not significant (*P *> 0.05) in multivariable models, which confirms that the associations were similar in normotensive and hypertensive subjects.

Table 3 presents regression coefficients of the *2677T *and the *3435T *allele, respectively, for the association with the renal haemodynamic phenotypes. Models adjusted for only age and sex differed little (i.e. by less than 30 percent of a standard error; *P *> 0.60) from complete multivariable models, which suggests that the extra covariate adjustment had little influence on the results. In multivariable models, each copy of the *2677T *allele was associated with a 10.6 mL/min higher GFR, a 47.5 mL/min higher ERPF, and a 0.016 lower RVR as compared to the GG reference genotype (P < 0.0001). While the effect sizes of the *2677T *allele were about 10% of the trait values, those of the *3435T *allele were smaller and less significant. These results suggest that, even after accounting for major known confounding factors, *ABCB1 *genetic variants were associated with renal function in this large sample. The results of sensitivity analyses including extreme outlier values did not differ by more than 1 standard error from the analyses conducted after excluding extreme outliers (*P *> 0.40).

**Table 3:** Association between the *2677T *and *3435T ABCB1 *alleles and renal haemodynamics in the Seychelles.

		**Dependent variable**								
		**GFR (mL/min)**			**ERPF (mL/min)**			**RVR (mm Hg/mL/min)**		

**Allele**	**Model**	**Coeff**	**SE**	**P value**	**Coeff**	**SE**	**P value**	**Coeff**	**SE**	**P value**

*2677T*	Model 1	9.8	3.5	0.005	37.7	14.3	0.008	-0.015	0.005	0.003
	Model 2	10.6	2.9	0.0002	47.5	11.6	0.00004	-0.016	0.004	0.0002
*3435T*	Model 1	6.1	2.7	0.03	29.5	11.4	0.009	-0.011	0.004	0.006
	Model 2	4.4	2.3	0.06	28.1	10.5	0.007	-0.011	0.004	0.004

Coeff: regression coefficient. SE: standard error. This table presents 3 * 4 different models, 4 for each dependent variable. Models 1 have age and sex as covariates, in addition to either the *2677T *or the *3435T *allele. Models 2 have age, sex, mean arterial blood pressure, diabetes mellitus, fasting blood glucose, body mass index, 24-h urine Na and K excretion (in mmol/24-h) as covariates. An additive mode of action was used for the T allele. All models are adjusted for ascertainment. Mean arterial pressure was not added as a covariate for RVR models.

Analyses restricted to subjects with at least 3 grand-parents of African descent showed results that were similar in direction and significance level to those obtained in the whole sample. There was also a trend toward a similar association of GFR estimated using MDRD with the *2677T *(8.6 ± 5.8 P = 0.14) and the *3435T *(10.1 ± 5.3, P = 0.058) alleles in analysis of MDRD in the 119 subjects for whom inulin clearance values were missing.

Haplotype analyses showed that each copy of the TT haplotype was associated with an 11.6 ml/min higher GFR, a 50.7 mL/min higher ERPF, and a 0.017 mm Hg/mL/min lower RVR than the reference GC haplotype (P < 0.001). These results suggest that most of the signal was carried by the *2677T *allele because the *3435T *allele adds only 1 mL/min GFR or 3 ml/min ERPF.

### CoLaus study

The 2526 men and 2856 women analyzed were aged 53.4 ± 10.7 years (mean ± SD), had mean 25.8 ± 4.5 kg/m2 BMI and 79.6 ± 14.6 μmol/L serum creatinine. The SNPs measured in the Seychelles (rs1045642 [*3435 C>T*], genotyped in CoLaus, and rs2032582 [*2677 G>T*], which is tagged by 3 genotyped SNPs at r^2 ^> 0.9 and 2 further genotyped SNPs at r^2 ^> 0.5) did not show any association with any estimate of GFR [see Additional file 2]. We did however find strong evidence of association between GFR and other SNPs in *ABCB1*. Testing the 30 directly genotyped SNPs within 10 kb of *ABCB1*, and using square-root transformed MDRD, we obtained P values of 0.0006, 0.0007, 0.0071, 0.0079 and 0.0085 for the five most significant SNPs, using 10^5 ^permutations. All other SNPs had P values > 0.01 [see Additional file 2]. The overall permutation P value for the most significant SNP, corrected for multiple testing, was P = 0.014. We take this as positive evidence for association.

The significance levels achieved here were sensitive to the removal of 25 outlying individuals (using a 4 SD cutoff). The overall permutation P value increased to 0.033 when only 13 outliers were excluded (using a 5 SD cutoff), and increased further to 0.10 (non-significant) when no outliers were excluded. Using other estimates of GFR, the associations were slightly less significant. For square-root transformed CG, P = 0.0007, 0.0018, 0.0079, 0.0046 and 0.0103 for the same top five SNPs, and P = 0.018 overall (i.e. corrected for multiple testing). For log transformed creatinine, P = 0.0009, 0.0010, 0.0060, 0.0069 and 0.0073 for the top five SNPs and P = 0.017 overall.

**Figure 3:**
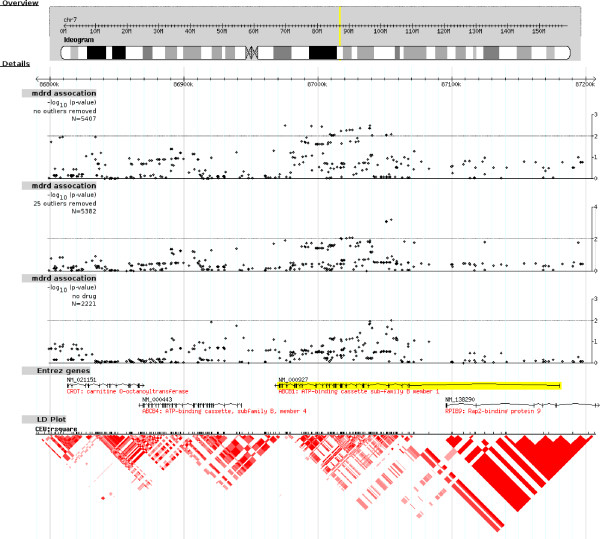
**Genetic associations with GFR, in the region around the *ABCB1 *gene**. Associations are shown for both directly genotyped and imputed SNPs. The bottom panel shows the pattern of linkage disequilibrium in the HapMap CEU panel. We imputed genotypes for all HapMap SNPs in the region around *ABCB1*. In this region, linkage disequilibrium patterns in CoLaus were similar to the ones observed in HapMap, although they were resolved at a coarser scale due to lower SNP density. Since it was not computationally feasible to combine imputation and permutation approaches, we plot P values calculated assuming the normal linear model. For directly genotyped SNPs, the differences between calculated and permutation P values were small. The top three hits (and P values) are rs17327624 (0.0006), rs4148733 (0.0008) and rs17327442 (0.0008). The former two were directly genotyped and are in strong LD (D' = 0.96 in CoLaus and 1.00 in HapMap CEU, r^2 ^= 0.63 and 0.72 respectively), and the imputed SNP rs17327442 is in perfect LD with rs17327624 in HapMap CEU.

To explore the possibility that SNPs not genotyped on the Affymetrix 500 K chip might be associated with GFR, we repeated our analyses using imputed SNP genotypes over a 400 kb region surrounding *ABCB1*. Results both with and without outlying individuals removed are shown in Figure 3. Three non-coding SNPs, including our top two hits listed above, showed strong associations (P < 0.001) [see Additional file 2].

In analyses restricted to 2224 individuals not taking any prescription drugs (excluding 3 outlying individuals at a 5 SD cutoff), 5 SNPs had permutation P values less than 0.05, and the overall permutation P value corrected for multiple testing was 0.11. Given the expected reduced power in this smaller sample size, we conclude that a similar association is observed in untreated subjects.

## Discussion

In the Seychelles' sample, we measured renal function using the accepted gold standard, inulin clearance, and found that *ABCB1 *genetic variants are associated with the haemodynamic profile, i.e. GFR, ERPF and RVR in these individuals of African descent with a positive family history of hypertension. We confirmed this association of the *ABCB1 *gene with renal function, estimated using the simplified MDRD equation, in a large population-based study of Caucasian subjects. This is, to our knowledge, the first time that the *ABCB1 *gene has been shown to be associated with renal function in the general population.

As this is not a true replication study (i.e. different *ABCB1 *marker variants were found to be associated with renal function in the two populations), these results are preliminary and need to be confirmed in other studies. This may result from the fact that the Seychelles and Swiss populations have different ethnic backgrounds and different linkage disequilibrium structures. In addition, the Seychelles sample was enriched in hypertensive individuals, whereas the Swiss one was not, which may further explain differences in findings between the two populations. We corrected our models for ascertainment (i.e. for the fact that recruitment was constrained to include two hypertensive siblings in each family). Such a correction aims to determine what the results would have been had the participants not been ascertained this way. This correction results in better generalizability of our observations.

In the African sample, (1) analyses restricted to subjects with at least 3 grand-parents of African descent led to very similar results; and (2) we confirmed the association with GFR estimated using MDRD in an analysis that included 119 additional African subjects. These results are thus unlikely to be due to population stratification. The confirmation of an association in the Caucasian sample with variants in the same gene adds further evidence against a spurious association.

The frequency of the *2677T *allele typically varies between 0–10% in subjects of African descent, 39–46% in Caucasians and 36–44% in Asians [24], whereas the frequency of the *3435T *allele typically varies between 16–27% in subjects of African descent, 48–57% in Caucasians, and 41–66% in Asians. [25] Of note, the frequencies of the *2677T *(31%) and *3435T *(54%) alleles in our sample are higher than those reported in other groups of African descent. The Seychelles' population is a relatively young population (the islands were first inhabited at the end of the 18^th ^century) of small size (currently 84,000 inhabitants) that has undergone a population bottleneck during the middle of the 19^th ^century followed by rapid growth (census data). Genetic drift may explain why the allele frequency differs in Seychelles versus other African populations (in Africa or elsewhere). It is not clear to what extent our observations may reflect the facts that homozygosity at the *3435T *allele is a marker of African descent and African ethnicity is a risk factor for developing chronic kidney disease. Given the large interethnic differences in allele frequencies observed for *ABCB1 *variants, it is important that the associations of *ABCB1 *variants with renal function are explored further in other ethnic groups.

The *TT *genotype of the *C3435T *polymorphism is associated with a significant reduction in PGP expression in various cell types, including renal proximal tubular cells. [26-29] Although synonymous (i.e. not leading to an amino-acid change in PGP), the *3435 C>T *variant influences mRNA expression by acting on its stability [30] and on the substrate specificity of PGP [31]. The *2677 G>T *variant is functional and leads to an Ala893Ser amino acid change in PGP. The *3435 C>T *and *2677 G>T *polymorphisms are in strong linkage disequilibrium. The fact that the *2677 G>T *and *3435 C>T ABCB1 *variants (and markers in strong linkage disequilibrium with these variants) were not associated with estimated GFR in the CoLaus study suggests that the true causal variant(s) is (are) in linkage disequilibrium with the variants genotyped in the Seychelles sample or that the two populations differ in either modifier genes or environmental components (e.g. the Balkanic toxin [32,33]) affecting the impact of the causal variant. Further studies are needed to clarify how *ABCB1 *genetic variants are associated with renal function.

The association reported here between the *ABCB1 *gene and renal haemodynamics does not necessarily mean a causal relationship. However, several independent lines of evidence of an association with renal function strengthen our confidence that these results are not spurious. First, several experimental findings *in vitro *and *in vivo *[5,8,9] and findings in humans [11,12] suggest that either PGP itself and/or *ABCB1 *genetic variants play a role in post-transplantation nephrotoxicity induced by calcineurin inhibitors, such as ciclosporine. Although inconsistent (i.e. the T allele is associated with nephrotoxicity in one study, but with nephroprotection in the other), these findings nevertheless suggest that PGP influences renal function via exogenous substances. Hence PGP may influence renal function via the control of intracellular concentration of a known toxin in the tubular epithelial cells. By analogy, we put could speculate that PGP influences renal function via the control of intracellular concentration of endogenous substances, by an as yet unknown mechanism. Second, Atanasova *et al *[32] found the *ABCB1 *haplotype 2677G/3435T to be protective against Balkan endemic nephropathy, a slowly progressive nephropathy associated with a high incidence of epithelial tumors of the upper urinary tract. Because of the very specific and stable geographical localization of this nephropathy, it has been proposed that an environmental toxin could play a role in its etiology. [33] However, the familial aggregation of this nephropathy suggests that genetic factors are also likely to be involved. [33] Third, endothelins are important regulators of renal blood flow and glomerular filtration rate [34] that can influence PGP-mediated transport [35,36]. Bosentan, an endothelin receptor antagonist, activates the pregnane X receptor [37], which itself activates *ABCB1 *expression [38].

## Conclusion

In conclusion, we found that selected *ABCB1 *gene variants were associated with renal function and haemodynamics in families of African descent, while other *ABCB1 *gene variants were associated with GFR estimates (MDRD and CG) in another study of Caucasian individuals. Although the associations were not based on the same markers in the two populations, these results suggest that variants of the *ABCB1 *gene influence renal function in the general population. The *ABCB1 *gene therefore represents a new candidate gene for nephropathy in humans. Replication in additional studies is warranted.

## Abbreviations

PGP: P-glycoprotein; GFR: glomerular filtration rate; ERPF: effective renal plasma flow; MDRD: Modification of the Diet in Renal Disease; CG: Cockcroft-Gault; RVR: renal vascular resistance; MAP: Mean arterial blood pressure; BMI; body mass index.

## Competing interests

The authors declare that they have no competing interests. Dawn Waterworth and Vincent Mooser are full-time employees of GlaxoSmithKline.

## Authors' contributions

AG, CBE, DMW, MBu, MM, PB, PV and VM participated to the conduct and/or design of the studies. MBo, JSB, RCE, TJ and SB participated in the data analyses. DMW, MBo, MM, PV, and VM were involved in the data acquisition. All authors have been involved in drafting the manuscript or revising it critically for important intellectual content. All authors have given final approval of the version to be published.

## Pre-publication history

The pre-publication history for this paper can be accessed here:



## Supplementary Material

Additional file 1 Additional information on methods. The data provided represent additional information on phenotype and covariate measurements in the Seychelles study and statistical analyses in the CoLaus study Click here for file

Additional file 2 Association of *ABCB1 *SNPs with square-root transformed MDRD in CoLaus. The data provided represent association data of genotyped and imputed SNPs located within and around the *ABCB1 *gene with square-root transformed MDRD in CoLaus. Click here for file
