# Supplementary material for: Association of ABCB1 genetic variants with renal function in Africans and in Caucasians
Source: BMC Med Genomics. 2008 Jun 2;1:21. doi: 10.1186/1755-8794-1-21 (PMC2424071; doi:10.1186/1755-8794-1-21)
Supplement: Additional file 1 — Additional information on methods. The data provided represent additional information on phenotype and covariate measurements in the Seychelles study and statistical analyses in the CoLaus study [file 1755-8794-1-21-S1.doc]

# Supplementary Appendix

# Phenotype and covariate measurements: Seychelles study

*Inulin and PAH clearances*

After overnight fasting, clearance protocols began between 7 and 8 am in a quiet room with the subject lying on a bed throughout the procedure except for active voiding. Two intravenous catheters were inserted into antecubital veins, one for the infusion of inulin and PAH, and the second into the contralateral forearm for blood drawing. Fasting blood samples were collected first. After an oral water load of 200 ml, a bolus and a following sustained infusion of inulin and PAH adapted to the participant’s height, weight and sex were given to ensure a stable plasma concentration. Participants received 400 ml of oral water after 60 minutes and thereafter 200 ml every hour.

# Statistical analyses: CoLaus study

In addition to the phenotypic covariates described in the main body of the paper, we controlled for population stratification by including as covariates the first 10 ancestry principal components (PCs) computed using the EIGENSTRAT methodology [1], and also counts of the number of grandparents born in each European country.

The three phenotypes of interest (creatinine, CG and MDRD), plus log and square-root transformed phenotypes, were regressed simultaneously onto all covariates. Visual examination of the residuals suggested that log(creatinine), square-root(CG) and square-root (MDRD) were close to normal in the bulk of the distribution, but had very heavy tails. We therefore excluded 25 individuals with extreme or outlying residuals, using a 4 SD cutoff for declaring outliers.

In our initial analyses to confirm the association, we used only directly genotyped SNPs. For each SNP, we regressed the corrected phenotype onto genotype, assuming additive effects for the SNP. Association tests were performed using PLINK (v0.99s, <http://pngu.mgh.harvard.edu/purcell/plink>) [2]. We obtained essentially identical results when we either (i) included simultaneously all covariates on a SNP by SNP basis, or (ii) first corrected the phenotype by regressing simultaneously onto all covariates (without any SNPs), and then used the residuals from that regression as a “corrected phenotype” for the association analysis. Analyses in which only “significant” covariates were included, gave very similar results. We used permutation tests (with 100,000 permutations) to compute P values for individual SNPs and also a global P value that is corrected for multiple testing. In further exploratory analyses, SNPs not directly genotyped were imputed using IMPUTE software [3], using reference haplotypes from HapMap release 21, on build 35 coordinates. Overall, 291 SNPs were imputed within and around the *ABCB1* gene. Final results were converted to build 36 coordinates; the two builds are collinear over the whole region of interest. Posterior distributions for the effect of each SNP (β) were calculated by averaging over 1000 random imputations of the genotypes at each SNP. We assumed diffuse normal-inverse-gamma priors. The reported P values are actually the smaller of 2 × Pr(β < 0 | data) and 2 × Pr(β >0 | data), which is exactly equivalent to the classical two tailed P value when there are no imputed or missing data. [4]

Associations were viewed in their genomic context using the HapMap Genome Browser ([http://hapmap.org](http://hapmap.org/)), using release 22 on build 36 coordinates.

Reference List

(1) Price AL, Patterson NJ, Plenge RM, Weinblatt ME, Shadick NA, Reich D: **Principal components analysis corrects for stratification in genome-wide association studies.** *Nat Genet* 2006, **38**:904-909.

(2) Purcell S, Neale B, Todd-Brown K, Thomas L, Ferreira MA, Bender D, Maller J, Sklar P, de Bakker PI, Daly MJ, Sham PC: **PLINK: a tool set for whole-genome association and population-based linkage analyses.** *Am J Hum Genet* 2007, **81**:559-575.

(3) Marchini J, Howie B, Myers S, McVean G, Donnelly P: **A new multipoint method for genome-wide association studies by imputation of genotypes.** *Nat Genet* 2007, **39**:906-913.

(4) O'Hagan A, Forster J. Bayesian Inference. In: Arnold, editor. Kendall's Advanced Theory of Statistics. 2nd ed. London: 2004.
