# Supplementary material for: Association of ABCB1 genetic variants with renal function in Africans and in Caucasians
Source: BMC Med Genomics. 2008 Jun 2;1:21. doi: 10.1186/1755-8794-1-21 (PMC2424071; doi:10.1186/1755-8794-1-21)
Supplement: Additional file 2 — Association of ABCB1 SNPs with square-root transformed MDRD in CoLaus. The data provided represent association data of genotyped and imputed SNPs located within and around the ABCB1 gene with square-root transformed MDRD in CoLaus. [file 1755-8794-1-21-S2.pdf]

Association with SQRT transformed MDRD

SNP rs number	position on build 36	allele1 (on + strand of reference sequence)	allele2	frequency of allele 2	number of observed genotypes	PLINK analysis: effect of allele 2 (beta)	PLINK analysis: SE for beta	PLINK analysis: P value	Permutation tests: P value for single SNP	Permutation tests: P value for all SNPs in ABCB1	Analysis of imputed genotypes: effect of allele 2 (beta)	Analysis of imputed genotypes: SE for beta	Analysis of imputed genotypes: P value
rs802031	86799298	C	T	0.2252	5331	0.0047	0.0233	0.8406	NA	NA	0.0015	0.0232	0.9500
rs11760687	86800677	C	T	0.9425	0	NA	NA	NA	NA	NA	-0.0597	0.0436	0.1708
rs11771466	86800739	A	G	0.9425	0	NA	NA	NA	NA	NA	-0.0601	0.0436	0.1683
rs802032	86801186	A	G	0.9504	0	NA	NA	NA	NA	NA	-0.0001	0.0455	0.9984
rs10499902	86802905	A	G	0.0147	0	NA	NA	NA	NA	NA	0.0364	0.0842	0.6662
rs11977186	86803101	A	C	0.0147	0	NA	NA	NA	NA	NA	0.0368	0.0843	0.6625
rs996745	86803535	C	T	0.6563	0	NA	NA	NA	NA	NA	-0.0139	0.0207	0.5019
rs802034	86803978	A	G	0.2248	0	NA	NA	NA	NA	NA	-0.0010	0.0233	0.9668
rs11975317	86804449	A	G	0.9853	0	NA	NA	NA	NA	NA	-0.0366	0.0842	0.6630
rs11975323	86804620	C	T	0.0147	0	NA	NA	NA	NA	NA	0.0371	0.0842	0.6598
rs11976069	86804854	C	T	0.0147	0	NA	NA	NA	NA	NA	0.0387	0.0841	0.6449
rs10447793	86805923	C	T	0.3289	0	NA	NA	NA	NA	NA	0.0116	0.0210	0.5815
rs17149389	86807374	C	T	0.0520	0	NA	NA	NA	NA	NA	0.0683	0.0464	0.1410
rs802035	86807495	C	T	0.7604	0	NA	NA	NA	NA	NA	-0.0019	0.0229	0.9333
rs17149393	86809490	C	G	0.0517	0	NA	NA	NA	NA	NA	0.0686	0.0465	0.1401
rs2373726	86809831	A	G	0.3438	0	NA	NA	NA	NA	NA	0.0137	0.0207	0.5094
rs11983873	86813202	A	G	0.9852	0	NA	NA	NA	NA	NA	-0.0375	0.0842	0.6564
rs11983904	86813500	C	G	0.0148	0	NA	NA	NA	NA	NA	0.0392	0.0840	0.6407
rs6943711	86815504	C	T	0.0149	0	NA	NA	NA	NA	NA	0.0376	0.0839	0.6542
rs6943724	86815584	A	G	0.0149	0	NA	NA	NA	NA	NA	0.0394	0.0842	0.6397
rs6944268	86815645	A	G	0.9851	0	NA	NA	NA	NA	NA	-0.0405	0.0836	0.6281
rs802036	86815830	C	T	0.9312	0	NA	NA	NA	NA	NA	0.0012	0.0382	0.9758
rs802037	86816283	A	C	0.2248	0	NA	NA	NA	NA	NA	-0.0018	0.0233	0.9374
rs11971666	86816490	C	T	0.9851	0	NA	NA	NA	NA	NA	-0.0396	0.0838	0.6367
rs11975766	86817278	A	G	0.0149	0	NA	NA	NA	NA	NA	0.0397	0.0835	0.6340
rs6954473	86817455	A	G	0.6560	0	NA	NA	NA	NA	NA	-0.0132	0.0207	0.5222
rs4728695	86819459	G	T	0.3440	0	NA	NA	NA	NA	NA	0.0133	0.0206	0.5193
rs802038	86819737	C	G	0.2248	0	NA	NA	NA	NA	NA	-0.0019	0.0233	0.9341
rs802041	86820873	G	T	0.7753	5311	-0.0053	0.0244	0.8292	NA	NA	0.0017	0.0233	0.9431
rs7808249	86821651	A	G	0.7043	5345	-0.0054	0.0214	0.7997	NA	NA	0.0036	0.0213	0.8641
rs802023	86823504	C	T	0.2249	0	NA	NA	NA	NA	NA	-0.0020	0.0233	0.9308
rs802024	86823655	C	T	0.0547	0	NA	NA	NA	NA	NA	-0.0128	0.0425	0.7638
rs802025	86824568	C	T	0.0547	0	NA	NA	NA	NA	NA	-0.0118	0.0426	0.7811
rs802026	86826975	A	G	0.8975	0	NA	NA	NA	NA	NA	-0.0349	0.0328	0.2874

Association with SQRT transformed MDRD

SNP rs number	position on build 36	allele1 (on + strand of reference sequence)	allele2	frequency of allele 2	number of observed genotypes	PLINK analysis: effect of allele 2 (beta)	PLINK analysis: SE for beta	PLINK analysis: P value	Permutation tests: P value for single SNP	Permutation tests: P value for all SNPs in ABCB1	Analysis of imputed genotypes: effect of allele 2 (beta)	Analysis of imputed genotypes: SE for beta	Analysis of imputed genotypes: P value
rs802028	86829611	C	T	0.1020	0	NA	NA	NA	NA	NA	0.0369	0.0328	0.2605
rs802030	86831487	C	G	0.1017	0	NA	NA	NA	NA	NA	0.0391	0.0329	0.2348
rs2051950	86833858	C	T	0.9213	5373	0.0544	0.0362	0.1332	NA	NA	-0.0556	0.0361	0.1240
rs7802658	86833935	C	T	0.1563	0	NA	NA	NA	NA	NA	0.0198	0.0272	0.4659
rs802054	86834425	A	G	0.9226	0	NA	NA	NA	NA	NA	0.0187	0.0375	0.6185
rs701339	86835099	C	T	0.0773	0	NA	NA	NA	NA	NA	-0.0175	0.0374	0.6394
rs10247098	86835630	A	C	0.1595	0	NA	NA	NA	NA	NA	0.0175	0.0269	0.5167
rs3789250	86836271	C	T	0.8403	0	NA	NA	NA	NA	NA	-0.0172	0.0269	0.5216
rs6966140	86836778	A	G	0.2184	0	NA	NA	NA	NA	NA	0.0115	0.0239	0.6292
rs7786781	86837801	C	T	0.9172	0	NA	NA	NA	NA	NA	-0.0432	0.0363	0.2339
rs802057	86838761	G	T	0.0772	0	NA	NA	NA	NA	NA	-0.0137	0.0371	0.7122
rs1089472	86840670	A	G	0.0825	0	NA	NA	NA	NA	NA	-0.0041	0.0355	0.9088
rs6949638	86841002	C	T	0.8391	0	NA	NA	NA	NA	NA	-0.0165	0.0267	0.5370
rs802062	86842217	G	T	0.0757	5299	-0.0119	0.0368	0.7458	NA	NA	-0.0107	0.0367	0.7712
rs802042	86844503	C	G	0.0826	0	NA	NA	NA	NA	NA	-0.0039	0.0354	0.9118
rs258962	86845115	A	G	0.9220	0	NA	NA	NA	NA	NA	-0.0012	0.0372	0.9736
rs258963	86845546	A	G	0.9174	0	NA	NA	NA	NA	NA	0.0039	0.0354	0.9127
rs258965	86845891	C	T	0.0816	5374	-0.0073	0.0356	0.8382	NA	NA	-0.0071	0.0356	0.8426
rs31627	86848140	A	G	0.0826	0	NA	NA	NA	NA	NA	-0.0037	0.0354	0.9163
rs31629	86848824	A	G	0.0827	0	NA	NA	NA	NA	NA	-0.0035	0.0354	0.9205
rs31632	86850351	C	T	0.9171	0	NA	NA	NA	NA	NA	0.0030	0.0354	0.9319
rs1202331	86852369	C	T	0.0833	0	NA	NA	NA	NA	NA	-0.0024	0.0355	0.9466
rs39606	86857112	A	G	0.9156	0	NA	NA	NA	NA	NA	-0.0005	0.0355	0.9885
rs7785206	86858959	C	G	0.9210	0	NA	NA	NA	NA	NA	-0.0343	0.0367	0.3495
rs31644	86859960	C	G	0.0851	0	NA	NA	NA	NA	NA	0.0018	0.0354	0.9585
rs2072207	86860532	C	T	0.1643	0	NA	NA	NA	NA	NA	0.0192	0.0265	0.4690
rs31645	86860917	C	T	0.0853	0	NA	NA	NA	NA	NA	0.0022	0.0353	0.9494
rs1608949	86861131	A	T	0.8356	0	NA	NA	NA	NA	NA	-0.0194	0.0266	0.4646
rs7801257	86862588	C	G	0.0166	0	NA	NA	NA	NA	NA	-0.0014	0.0946	0.9867
rs17149512	86866095	C	T	0.0793	0	NA	NA	NA	NA	NA	0.0336	0.0365	0.3575
rs31651	86867223	A	G	0.9103	5357	0.0091	0.0350	0.7952	NA	NA	-0.0107	0.0340	0.7523
rs31652	86867623	A	G	0.1399	0	NA	NA	NA	NA	NA	0.0078	0.0304	0.7975
rs6465112	86867920	C	T	0.8298	0	NA	NA	NA	NA	NA	-0.0204	0.0257	0.4270
rs2097937	86868839	A	G	0.1703	5366	0.0199	0.0258	0.4404	NA	NA	0.0202	0.0257	0.4316

Association with SQRT transformed MDRD

SNP rs number	position on build 36	allele1 (on + strand of reference sequence)	allele2	frequency of allele 2	number of observed genotypes	PLINK analysis: effect of allele 2 (beta)	PLINK analysis: SE for beta	PLINK analysis: P value	Permutation tests: P value for single SNP	Permutation tests: P value for all SNPs in ABCB1	Analysis of imputed genotypes: effect of allele 2 (beta)	Analysis of imputed genotypes: SE for beta	Analysis of imputed genotypes: P value
rs1526090	86869745	A	G	0.8298	0	NA	NA	NA	NA	NA	-0.0204	0.0257	0.4270
rs31653	86870549	A	G	0.9101	0	NA	NA	NA	NA	NA	-0.0064	0.0339	0.8495
rs12154399	86871884	C	T	0.1702	0	NA	NA	NA	NA	NA	0.0205	0.0257	0.4252
rs10487804	86874041	A	G	0.9198	0	NA	NA	NA	NA	NA	-0.0329	0.0360	0.3596
rs2373593	86874878	G	T	0.9102	0	NA	NA	NA	NA	NA	-0.0070	0.0339	0.8365
rs17149539	86876010	A	G	0.0802	0	NA	NA	NA	NA	NA	0.0330	0.0360	0.3580
rs31658	86877077	C	T	0.0895	5317	0.0149	0.0341	0.6615	NA	NA	0.0117	0.0340	0.7314
rs31659	86878565	C	T	0.9104	5322	0.0002	0.0340	0.9964	NA	NA	0.0013	0.0339	0.9694
rs11761050	86879792	A	C	0.9200	0	NA	NA	NA	NA	NA	-0.0351	0.0360	0.3297
rs17149547	86881166	A	G	0.0800	0	NA	NA	NA	NA	NA	0.0350	0.0360	0.3307
rs7807638	86881519	A	G	0.0800	0	NA	NA	NA	NA	NA	0.0352	0.0360	0.3290
rs4148830	86881644	A	T	0.1701	0	NA	NA	NA	NA	NA	0.0206	0.0258	0.4237
rs31660	86882379	A	G	0.9099	0	NA	NA	NA	NA	NA	-0.0046	0.0339	0.8924
rs31662	86883520	A	G	0.9100	5381	0.0040	0.0339	0.9052	NA	NA	-0.0038	0.0339	0.9106
rs31663	86883773	A	G	0.0187	0	NA	NA	NA	NA	NA	0.0241	0.0773	0.7552
rs4148829	86884406	A	G	0.0799	0	NA	NA	NA	NA	NA	0.0348	0.0361	0.3346
rs7788404	86885749	C	T	0.9201	0	NA	NA	NA	NA	NA	-0.0351	0.0361	0.3317
rs4148828	86886466	A	G	0.1111	0	NA	NA	NA	NA	NA	-0.0137	0.0365	0.7063
rs31666	86890204	C	T	0.8493	0	NA	NA	NA	NA	NA	-0.0556	0.0274	0.0430
rs6957497	86890344	C	T	0.0104	0	NA	NA	NA	NA	NA	-0.1053	0.1145	0.3568
rs6977539	86890415	C	T	0.9211	0	NA	NA	NA	NA	NA	-0.0373	0.0366	0.3073
rs6957680	86890541	A	G	0.0789	0	NA	NA	NA	NA	NA	0.0375	0.0366	0.3053
rs31667	86891086	A	G	0.8489	0	NA	NA	NA	NA	NA	-0.0554	0.0273	0.0428
rs31668	86891142	A	G	0.0723	0	NA	NA	NA	NA	NA	0.0663	0.0377	0.0788
rs31669	86892183	A	G	0.9236	5289	0.0677	0.0368	0.0658	NA	NA	-0.0627	0.0367	0.0873
rs11768699	86893571	G	T	0.0787	0	NA	NA	NA	NA	NA	0.0376	0.0365	0.3025
rs1017054	86893947	C	T	0.9213	0	NA	NA	NA	NA	NA	-0.0374	0.0365	0.3056
rs8187799	86894112	C	T	0.9213	0	NA	NA	NA	NA	NA	-0.0376	0.0365	0.3023
rs6956661	86895635	A	G	0.9213	0	NA	NA	NA	NA	NA	-0.0372	0.0365	0.3071
rs31670	86897100	A	G	0.2075	5369	0.0471	0.0237	0.0468	NA	NA	0.0452	0.0236	0.0558
rs31671	86897339	C	G	0.2075	5348	0.0467	0.0236	0.0484	NA	NA	0.0452	0.0236	0.0558
rs31672	86897635	C	T	0.8712	0	NA	NA	NA	NA	NA	-0.0433	0.0287	0.1316
rs6977739	86899470	G	T	0.0788	5378	0.0373	0.0362	0.3031	NA	NA	0.0368	0.0362	0.3091
rs17651890	86903623	A	G	0.9194	0	NA	NA	NA	NA	NA	0.0319	0.0451	0.4773

Association with SQRT transformed MDRD

SNP rs number	position on build 36	allele1 (on + strand of reference sequence)	allele2	frequency of allele 2	number of observed genotypes	PLINK analysis: effect of allele 2 (beta)	PLINK analysis: SE for beta	PLINK analysis: P value	Permutation tests: P value for single SNP	Permutation tests: P value for all SNPs in ABCB1	Analysis of imputed genotypes: effect of allele 2 (beta)	Analysis of imputed genotypes: SE for beta	Analysis of imputed genotypes: P value
rs31674	86906400	C	T	0.2071	0	NA	NA	NA	NA	NA	0.0443	0.0237	0.0613
rs31675	86907694	C	T	0.0723	5311	0.0513	0.0383	0.1807	NA	NA	0.0540	0.0375	0.1500
rs31676	86907816	C	T	0.2070	0	NA	NA	NA	NA	NA	0.0441	0.0237	0.0628
rs17149601	86910162	C	T	0.0784	0	NA	NA	NA	NA	NA	0.0360	0.0365	0.3231
rs17149606	86910441	A	C	0.9216	0	NA	NA	NA	NA	NA	-0.0357	0.0365	0.3272
rs17149608	86911530	A	G	0.9238	0	NA	NA	NA	NA	NA	-0.0463	0.0393	0.2391
rs1149222	86911711	G	T	0.8142	0	NA	NA	NA	NA	NA	-0.0203	0.0266	0.4456
rs4148826	86912355	C	T	0.8877	0	NA	NA	NA	NA	NA	-0.0213	0.0325	0.5120
rs1468615	86912900	C	T	0.8880	0	NA	NA	NA	NA	NA	-0.0197	0.0326	0.5455
rs4148824	86913298	A	G	0.1753	0	NA	NA	NA	NA	NA	0.0070	0.0260	0.7865
rs4148823	86914080	C	T	0.0367	0	NA	NA	NA	NA	NA	-0.0336	0.0524	0.5213
rs1014283	86914523	A	C	0.8247	0	NA	NA	NA	NA	NA	-0.0067	0.0259	0.7962
rs4148822	86915932	A	G	0.0683	0	NA	NA	NA	NA	NA	0.0506	0.0408	0.2152
rs1558375	86917005	C	T	0.8248	0	NA	NA	NA	NA	NA	-0.0065	0.0258	0.8018
rs1558376	86917039	A	T	0.8248	0	NA	NA	NA	NA	NA	-0.0065	0.0258	0.8004
rs2109505	86917342	A	T	0.8249	0	NA	NA	NA	NA	NA	-0.0058	0.0257	0.8227
rs4148821	86917538	C	G	0.8249	0	NA	NA	NA	NA	NA	-0.0056	0.0257	0.8282
rs1473152	86918286	A	C	0.1751	0	NA	NA	NA	NA	NA	0.0059	0.0257	0.8188
rs2072383	86919285	A	G	0.1382	0	NA	NA	NA	NA	NA	0.0176	0.0286	0.5375
rs1202283	86920228	A	G	0.4588	5363	0.0378	0.0192	0.0493	0.0484	0.5378	0.0370	0.0192	0.0535
rs2072384	86920804	A	T	0.1747	0	NA	NA	NA	NA	NA	0.0048	0.0257	0.8508
rs17149647	86924867	C	T	0.8622	0	NA	NA	NA	NA	NA	-0.0168	0.0285	0.5555
rs4148817	86925333	C	G	0.1378	0	NA	NA	NA	NA	NA	0.0166	0.0285	0.5593
rs4148816	86925667	A	G	0.8254	0	NA	NA	NA	NA	NA	-0.0048	0.0257	0.8501
rs4148815	86925770	A	T	0.1378	0	NA	NA	NA	NA	NA	0.0168	0.0285	0.5545
rs4148814	86929479	C	T	0.8623	0	NA	NA	NA	NA	NA	-0.0167	0.0285	0.5571
rs2302385	86929762	C	T	0.8623	0	NA	NA	NA	NA	NA	-0.0168	0.0285	0.5565
rs2302386	86929880	A	G	0.1376	0	NA	NA	NA	NA	NA	0.0165	0.0285	0.5633
rs2302387	86930121	A	G	0.8636	0	NA	NA	NA	NA	NA	-0.0157	0.0285	0.5823
rs4148813	86930312	A	G	0.1732	0	NA	NA	NA	NA	NA	0.0032	0.0256	0.8997
rs988448	86931183	C	T	0.9633	0	NA	NA	NA	NA	NA	0.0397	0.0519	0.4441
rs4148812	86939343	C	G	0.3005	0	NA	NA	NA	NA	NA	0.0447	0.0220	0.0417
rs4148810	86940187	C	T	0.1264	0	NA	NA	NA	NA	NA	-0.0211	0.0301	0.4831
rs4148809	86941199	C	T	0.5277	0	NA	NA	NA	NA	NA	-0.0382	0.0199	0.0547

Association with SQRT transformed MDRD

						PLINK analysis			Permutation tests		Analysis of imputed genotypes		
SNP rs number	position on build 36	allele1 (on + strand of reference sequence)	allele2 (on + strand of reference sequence)	frequency of allele 2	number of observed genotypes	effect of allele 2 (beta)	SE for beta	P value	P value for single SNP	P value for all SNPs in ABCB1	effect of allele 2 (beta)	SE for beta	P value
rs2888611	86941606	C	G	0.1713	5378	0.0023	0.0256	0.9298	0.9299	1.0000	0.0033	0.0256	0.8987
rs2071645	86943212	C	G	0.8287	0	NA	NA	NA	NA	NA	0.0014	0.0257	0.9559
rs3747806	86944638	A	G	0.0365	5275	-0.0571	0.0538	0.2889	0.2877	0.9913	-0.0419	0.0521	0.4216
rs12539936	86947953	A	G	0.8285	5293	-0.0105	0.0258	0.6850	0.6864	1.0000	0.0054	0.0256	0.8333
rs12673662	86948287	C	G	0.1346	0	NA	NA	NA	NA	NA	0.0102	0.0286	0.7203
rs6465116	86950937	A	G	0.8656	0	NA	NA	NA	NA	NA	-0.0108	0.0286	0.7069
rs2178658	86959958	G	T	0.3038	0	NA	NA	NA	NA	NA	0.0439	0.0221	0.0472
rs7789645	86960539	C	G	0.8295	0	NA	NA	NA	NA	NA	0.0024	0.0258	0.9248
rs7793196	86960783	A	G	0.1704	0	NA	NA	NA	NA	NA	-0.0027	0.0258	0.9177
rs998671	86961211	C	T	0.1340	0	NA	NA	NA	NA	NA	0.0112	0.0288	0.6979
rs17209837	86962758	C	T	0.8663	0	NA	NA	NA	NA	NA	-0.0115	0.0289	0.6908
rs12672720	86964374	A	G	0.1335	0	NA	NA	NA	NA	NA	0.0119	0.0290	0.6825
rs6946119	86966801	C	T	0.7244	0	NA	NA	NA	NA	NA	-0.0513	0.0220	0.0194
rs7802783	86968333	C	T	0.1324	0	NA	NA	NA	NA	NA	0.0124	0.0294	0.6718
rs1055302	86970852	C	T	0.1198	0	NA	NA	NA	NA	NA	0.0141	0.0310	0.6497
rs17064	86971406	A	T	0.9344	0	NA	NA	NA	NA	NA	0.0212	0.0416	0.6113
rs6979885	86975397	A	G	0.7455	0	NA	NA	NA	NA	NA	-0.0608	0.0244	0.0123
rs6946379	86976122	A	G	0.9710	0	NA	NA	NA	NA	NA	-0.0046	0.0757	0.9502
rs2235047	86976468	T	G	0.0168	5376	-0.0850	0.0756	0.2607	0.2605	0.9854	NA	NA	NA
**rs1045642**	**86976581**	**T**	**C**	**0.4841**	**5382**	**0.0012**	**0.0191**	**0.9497**	**0.9495**	**1.0000**	**NA**	**NA**	**NA**
rs4437575	86977252	A	G	0.4633	0	NA	NA	NA	NA	NA	0.0159	0.0209	0.4447
rs1002205	86979110	C	G	0.9939	0	NA	NA	NA	NA	NA	0.0356	0.1704	0.8363
rs1002204	86979433	A	C	0.4871	0	NA	NA	NA	NA	NA	0.0107	0.0203	0.5982
rs17149699	86979687	C	T	0.0387	0	NA	NA	NA	NA	NA	-0.0502	0.0499	0.3143
rs6949448	86979750	C	T	0.4356	0	NA	NA	NA	NA	NA	-0.0078	0.0197	0.6928
rs4148751	86981089	C	T	0.9588	5219	-0.0395	0.0482	0.4128	0.4119	0.9996	0.0386	0.0481	0.4221
rs4148750	86981211	C	T	0.9592	5350	-0.0390	0.0487	0.4227	0.4218	0.9997	0.0364	0.0482	0.4503
rs1922243	86981440	C	T	0.0393	0	NA	NA	NA	NA	NA	-0.0492	0.0493	0.3183
rs4148749	86982349	C	G	0.9785	0	NA	NA	NA	NA	NA	0.0189	0.0678	0.7802
rs7779562	86982752	C	G	0.9593	5380	-0.0395	0.0483	0.4133	0.4105	0.9996	0.0392	0.0482	0.4159
rs4148745	86986201	G	T	0.0210	0	NA	NA	NA	NA	NA	-0.0210	0.0682	0.7582
rs2235067	86987858	C	T	0.1387	0	NA	NA	NA	NA	NA	-0.0607	0.0278	0.0287
rs4148744	86988710	A	G	0.9617	0	NA	NA	NA	NA	NA	0.0440	0.0502	0.3807
rs4148743	86989026	C	T	0.4709	5377	-0.0149	0.0190	0.4331	0.4312	0.9998	-0.0145	0.0190	0.4447

Association with SQRT transformed MDRD

SNP rs number	position on build 36	allele1 (on + strand of reference sequence)	allele2	frequency of allele 2	number of observed genotypes	PLINK analysis: effect of allele 2 (beta)	PLINK analysis: SE for beta	PLINK analysis: P value	Permutation tests: P value for single SNP	Permutation tests: P value for all SNPs in ABCB1	Analysis of imputed genotypes: effect of allele 2 (beta)	Analysis of imputed genotypes: SE for beta	Analysis of imputed genotypes: P value
rs4148740	86990039	A	G	0.1387	0	NA	NA	NA	NA	NA	-0.0603	0.0277	0.0297
rs11979702	86990559	A	T	0.8613	0	NA	NA	NA	NA	NA	0.0596	0.0277	0.0313
rs2373588	86991096	A	G	0.9620	0	NA	NA	NA	NA	NA	0.0451	0.0504	0.3708
rs10280101	86991521	A	C	0.1406	4976	-0.0704	0.0284	0.0131	0.0133	0.2015	-0.0648	0.0275	0.0185
rs10225473	86992582	A	G	0.1386	0	NA	NA	NA	NA	NA	-0.0589	0.0277	0.0332
rs7787082	86994987	A	G	0.8246	0	NA	NA	NA	NA	NA	0.0594	0.0251	0.0181
rs2373587	86995402	C	G	0.0375	0	NA	NA	NA	NA	NA	-0.0469	0.0506	0.3541
rs2373586	86995519	A	C	0.5673	0	NA	NA	NA	NA	NA	0.0117	0.0193	0.5445
rs2032583	86998497	A	G	0.1377	0	NA	NA	NA	NA	NA	-0.0579	0.0277	0.0364
**rs2032582**	**86998554**	**A**	**C**	**0.5672**	**0**	**NA**	**NA**	**NA**	**NA**	**NA**	**0.0119**	**0.0193**	**0.5388**
rs4148739	86998985	C	T	0.8623	0	NA	NA	NA	NA	NA	0.0580	0.0277	0.0363
rs11983225	86999456	C	T	0.8623	0	NA	NA	NA	NA	NA	0.0579	0.0277	0.0366
rs11760837	87000952	C	T	0.8624	0	NA	NA	NA	NA	NA	0.0578	0.0277	0.0369
rs4148738	87000985	C	T	0.5485	5372	-0.0152	0.0192	0.4280	0.4259	0.9997	0.0169	0.0191	0.3776
rs10274587	87002419	A	G	0.8624	0	NA	NA	NA	NA	NA	0.0577	0.0277	0.0372
rs10234411	87002828	A	T	0.4328	0	NA	NA	NA	NA	NA	-0.0120	0.0193	0.5349
rs10248420	87002922	A	G	0.1750	0	NA	NA	NA	NA	NA	-0.0592	0.0251	0.0185
rs2235043	87003633	A	G	0.9840	0	NA	NA	NA	NA	NA	-0.0924	0.0864	0.2845
rs2235040	87003686	C	T	0.1376	5375	-0.0564	0.0277	0.0419	0.0423	0.4845	-0.0577	0.0277	0.0373
rs12668877	87004940	C	T	0.0204	0	NA	NA	NA	NA	NA	-0.0250	0.0683	0.7143
rs3789246	87005963	C	T	0.0204	0	NA	NA	NA	NA	NA	-0.0250	0.0683	0.7146
rs4728699	87006685	C	T	0.9608	0	NA	NA	NA	NA	NA	0.0024	0.0638	0.9691
rs7795817	87006973	C	T	0.0208	5327	-0.0375	0.0698	0.5906	0.5914	1.0000	-0.0235	0.0676	0.7279
rs12720067	87007292	C	T	0.1359	5133	-0.0539	0.0290	0.0633	0.0628	0.6232	-0.0559	0.0280	0.0461
rs10268314	87007605	C	T	0.8627	0	NA	NA	NA	NA	NA	0.0601	0.0277	0.0300
rs12720066	87007638	A	C	0.0893	0	NA	NA	NA	NA	NA	-0.0355	0.0428	0.4087
rs2235063	87008743	C	T	0.9938	0	NA	NA	NA	NA	NA	0.0473	0.1656	0.7798
rs4148737	87009088	C	T	0.5864	0	NA	NA	NA	NA	NA	-0.0441	0.0194	0.0230
rs10276603	87009463	C	T	0.8624	0	NA	NA	NA	NA	NA	0.0600	0.0277	0.0303
rs4728700	87009595	C	T	0.4486	0	NA	NA	NA	NA	NA	-0.0138	0.0192	0.4712
rs4728701	87009989	A	G	0.9609	0	NA	NA	NA	NA	NA	0.0039	0.0635	0.9471
rs6961419	87010072	C	T	0.5861	0	NA	NA	NA	NA	NA	-0.0432	0.0194	0.0259
rs6980101	87010534	C	T	0.0207	0	NA	NA	NA	NA	NA	-0.0206	0.0685	0.7634
rs4148735	87010817	C	T	0.4138	5376	0.0434	0.0194	0.0250	0.0254	0.3353	0.0431	0.0193	0.0258

Association with SQRT transformed MDRD

SNP rs number	position on build 36	allele1 (on + strand of reference sequence)	allele2	frequency of allele 2	number of observed genotypes	PLINK analysis: effect of allele 2 (beta)	PLINK analysis: SE for beta	PLINK analysis: P value	Permutation tests: P value for single SNP	Permutation tests: P value for all SNPs in ABCB1	Analysis of imputed genotypes: effect of allele 2 (beta)	Analysis of imputed genotypes: SE for beta	Analysis of imputed genotypes: P value
rs1922242	87011603	A	T	0.4138	0	NA	NA	NA	NA	NA	0.0430	0.0194	0.0266
rs2235046	87012002	C	T	0.4485	0	NA	NA	NA	NA	NA	-0.0135	0.0192	0.4838
rs2091766	87012440	C	T	0.3873	0	NA	NA	NA	NA	NA	0.0452	0.0198	0.0226
rs10808072	87014399	A	G	0.4978	5377	0.0181	0.0190	0.3405	0.3382	0.9971	0.0174	0.0190	0.3582
rs2235013	87016562	C	T	0.4979	0	NA	NA	NA	NA	NA	0.0172	0.0190	0.3649
rs2235035	87017022	A	G	0.6750	5358	0.0546	0.0205	0.0078	0.0082	0.1298	-0.0533	0.0205	0.0093
rs2235033	87017079	A	G	0.4980	0	NA	NA	NA	NA	NA	0.0172	0.0190	0.3648
rs2032588	87017379	A	G	0.9464	0	NA	NA	NA	NA	NA	0.0193	0.0426	0.6500
rs1128503	87017537	A	G	0.5639	0	NA	NA	NA	NA	NA	0.0243	0.0193	0.2084
rs2229109	87017745	C	T	0.0636	0	NA	NA	NA	NA	NA	0.1172	0.0452	0.0096
rs10276036	87018134	C	T	0.5632	4943	-0.0204	0.0198	0.3020	0.3000	0.9933	0.0235	0.0193	0.2220
rs4728702	87018614	A	T	0.5639	0	NA	NA	NA	NA	NA	0.0245	0.0193	0.2052
rs12704364	87019111	C	T	0.4979	0	NA	NA	NA	NA	NA	0.0176	0.0190	0.3559
rs6961665	87019354	A	C	0.5021	0	NA	NA	NA	NA	NA	-0.0176	0.0190	0.3548
rs3789244	87019785	G	T	0.5703	0	NA	NA	NA	NA	NA	0.0239	0.0195	0.2201
rs1922240	87021290	C	T	0.6761	5382	0.0535	0.0205	0.0091	0.0092	0.1483	-0.0534	0.0205	0.0091
rs1922241	87023830	A	G	0.6757	0	NA	NA	NA	NA	NA	-0.0540	0.0206	0.0086
rs11772987	87023954	C	G	0.1378	0	NA	NA	NA	NA	NA	-0.0611	0.0278	0.0282
rs13239201	87025931	C	T	0.6759	0	NA	NA	NA	NA	NA	-0.0541	0.0206	0.0086
rs10244266	87026403	G	T	0.8604	0	NA	NA	NA	NA	NA	0.0558	0.0293	0.0571
rs12154319	87026493	A	G	0.9914	0	NA	NA	NA	NA	NA	-0.0328	0.1187	0.7833
rs868755	87027866	G	T	0.4226	0	NA	NA	NA	NA	NA	-0.0204	0.0196	0.2976
rs1882479	87028022	A	G	0.1373	0	NA	NA	NA	NA	NA	-0.0610	0.0280	0.0296
rs2235023	87028388	C	T	0.0648	0	NA	NA	NA	NA	NA	0.0276	0.0396	0.4861
rs13237132	87029605	C	G	0.3167	0	NA	NA	NA	NA	NA	0.0494	0.0210	0.0185
rs956825	87030211	C	T	0.3233	0	NA	NA	NA	NA	NA	0.0468	0.0211	0.0268
rs11975994	87030667	A	G	0.4398	0	NA	NA	NA	NA	NA	-0.0250	0.0195	0.2004
rs4148734	87031533	A	G	0.7043	0	NA	NA	NA	NA	NA	-0.0515	0.0213	0.0157
rs1202170	87033042	C	T	0.5121	0	NA	NA	NA	NA	NA	-0.0142	0.0195	0.4645
rs1202169	87033786	C	T	0.5579	0	NA	NA	NA	NA	NA	0.0253	0.0196	0.1962
rs1202167	87034995	C	T	0.4443	0	NA	NA	NA	NA	NA	-0.0256	0.0196	0.1923
rs1024409	87036303	A	G	0.9734	0	NA	NA	NA	NA	NA	-0.0006	0.0594	0.9937
rs2235019	87037239	A	C	0.9878	0	NA	NA	NA	NA	NA	-0.2156	0.0882	0.0147
rs2235015	87037500	A	C	0.8193	0	NA	NA	NA	NA	NA	0.0368	0.0274	0.1793

Association with SQRT transformed MDRD

SNP rs number	position on build 36	allele1 (on + strand of reference sequence)	allele2	frequency of allele 2	number of observed genotypes	PLINK analysis: effect of allele 2 (beta)	PLINK analysis: SE for beta	PLINK analysis: P value	Permutation tests: P value for single SNP	Permutation tests: P value for all SNPs in ABCB1	Analysis of imputed genotypes: effect of allele 2 (beta)	Analysis of imputed genotypes: SE for beta	Analysis of imputed genotypes: P value
rs6950978	87038403	A	T	0.2914	0	NA	NA	NA	NA	NA	0.0521	0.0216	0.0158
rs10256836	87038709	C	G	0.7099	0	NA	NA	NA	NA	NA	-0.0539	0.0216	0.0124
rs10259849	87038778	C	T	0.7089	0	NA	NA	NA	NA	NA	-0.0522	0.0216	0.0155
rs2520464	87039022	C	T	0.4445	0	NA	NA	NA	NA	NA	-0.0265	0.0197	0.1789
rs12334183	87039316	C	T	0.8114	0	NA	NA	NA	NA	NA	0.0464	0.0252	0.0660
rs10260862	87039418	C	G	0.8168	0	NA	NA	NA	NA	NA	0.0461	0.0257	0.0727
rs10280623	87040480	C	T	0.8133	0	NA	NA	NA	NA	NA	0.0462	0.0252	0.0667
rs10264990	87040551	C	T	0.6654	5346	0.0463	0.0202	0.0222	0.0226	0.3068	-0.0437	0.0202	0.0305
rs1202180	87041776	C	T	0.6713	0	NA	NA	NA	NA	NA	0.0225	0.0208	0.2789
rs1202179	87042215	C	T	0.6717	0	NA	NA	NA	NA	NA	0.0224	0.0208	0.2818
rs1989831	87043415	A	T	0.3282	0	NA	NA	NA	NA	NA	-0.0222	0.0208	0.2848
rs1989830	87043599	A	G	0.6719	0	NA	NA	NA	NA	NA	0.0223	0.0208	0.2827
rs13226726	87044551	C	T	0.1120	0	NA	NA	NA	NA	NA	-0.0396	0.0381	0.2971
rs1202175	87047086	A	G	0.3278	0	NA	NA	NA	NA	NA	-0.0222	0.0207	0.2856
rs1202174	87047308	C	T	0.3278	0	NA	NA	NA	NA	NA	-0.0221	0.0207	0.2858
rs1202172	87048910	A	C	0.3276	0	NA	NA	NA	NA	NA	-0.0221	0.0207	0.2858
rs1202171	87048981	A	T	0.3275	5341	-0.0226	0.0207	0.2760	0.2757	0.9891	-0.0213	0.0207	0.3031
rs17327442	87050926	A	T	0.8433	0	NA	NA	NA	NA	NA	-0.0881	0.0264	0.0008
rs4148733	87051168	A	G	0.1568	5358	0.0898	0.0264	0.0007	0.0008	0.0143	0.0887	0.0264	0.0008
rs1202186	87051194	C	T	0.6725	0	NA	NA	NA	NA	NA	0.0222	0.0207	0.2837
rs1202185	87051320	C	T	0.6725	0	NA	NA	NA	NA	NA	0.0222	0.0207	0.2834
rs1202184	87051837	C	T	0.5079	0	NA	NA	NA	NA	NA	-0.0275	0.0194	0.1554
rs1211152	87053050	A	C	0.9445	5271	0.0131	0.0443	0.7679	0.7663	1.0000	-0.0149	0.0429	0.7287
rs1202182	87053240	A	G	0.3275	0	NA	NA	NA	NA	NA	-0.0223	0.0208	0.2830
rs1202181	87054086	A	G	0.3275	0	NA	NA	NA	NA	NA	-0.0224	0.0208	0.2817
rs17327624	87054753	G	T	0.2142	5148	0.0823	0.0244	0.0008	0.0008	0.0161	0.0811	0.0237	0.0006
rs11763872	87055151	C	T	0.4771	0	NA	NA	NA	NA	NA	0.0305	0.0198	0.1244
rs7802773	87056283	A	G	0.4978	0	NA	NA	NA	NA	NA	-0.0262	0.0204	0.1993
rs13229143	87057417	C	G	0.4633	0	NA	NA	NA	NA	NA	-0.0028	0.0216	0.8974
rs12535512	87058270	C	T	0.5792	0	NA	NA	NA	NA	NA	0.0188	0.0208	0.3674
rs2188526	87058498	C	T	0.4602	0	NA	NA	NA	NA	NA	-0.0196	0.0208	0.3466
rs3789243	87058822	A	G	0.4829	0	NA	NA	NA	NA	NA	-0.0235	0.0205	0.2517
rs1858923	87059152	A	G	0.4601	0	NA	NA	NA	NA	NA	-0.0194	0.0207	0.3483
rs17149792	87062187	C	T	0.0230	0	NA	NA	NA	NA	NA	-0.0570	0.0673	0.3966

Association with SQRT transformed MDRD

SNP rs number	position on build 36	allele1 (on + strand of reference sequence)	allele2	frequency of allele 2	number of observed genotypes	PLINK analysis: effect of allele 2 (beta)	SE for beta	P value	Permutation tests: P value for single SNP	P value for all SNPs in ABCB1	Analysis of imputed genotypes: effect of allele 2 (beta)	SE for beta	P value
rs2188525	87062708	A	C	0.9604	0	NA	NA	NA	NA	NA	0.0086	0.0510	0.8656
rs2214103	87062884	C	G	0.0396	0	NA	NA	NA	NA	NA	-0.0082	0.0508	0.8714
rs2235074	87062982	A	G	0.9604	0	NA	NA	NA	NA	NA	0.0083	0.0509	0.8710
rs17327942	87064626	A	G	0.0121	0	NA	NA	NA	NA	NA	0.2258	0.0921	0.0144
rs2888599	87066961	C	T	0.0397	0	NA	NA	NA	NA	NA	-0.0089	0.0508	0.8607
rs9282564	87067376	C	T	0.8983	0	NA	NA	NA	NA	NA	0.0396	0.0402	0.3237
rs2214102	87067437	C	T	0.0801	0	NA	NA	NA	NA	NA	0.0408	0.0386	0.2909
rs3213619	87068129	A	G	0.0397	0	NA	NA	NA	NA	NA	-0.0092	0.0507	0.8565
rs4728709	87071538	A	G	0.9352	5356	-0.0234	0.0394	0.5533	0.5522	1.0000	0.0248	0.0392	0.5264
rs4148732	87071985	C	T	0.8609	0	NA	NA	NA	NA	NA	-0.0680	0.0285	0.0171
rs4148731	87077265	A	G	0.9761	0	NA	NA	NA	NA	NA	0.0629	0.0654	0.3363
rs4148730	87077287	A	G	0.0239	0	NA	NA	NA	NA	NA	-0.0623	0.0653	0.3396
rs13233308	87082896	C	T	0.4657	0	NA	NA	NA	NA	NA	-0.0172	0.0208	0.4073
rs10231033	87099516	A	G	0.0262	0	NA	NA	NA	NA	NA	-0.0620	0.0639	0.3320
rs10276499	87099672	C	T	0.9362	0	NA	NA	NA	NA	NA	0.0325	0.0396	0.4119
rs10264856	87100517	A	G	0.9358	5352	-0.0360	0.0394	0.3604	0.3590	0.9983	0.0325	0.0393	0.4083
rs17250003	87100659	A	G	0.0307	0	NA	NA	NA	NA	NA	-0.0373	0.0597	0.5315
rs17149840	87100879	A	G	0.9738	0	NA	NA	NA	NA	NA	0.0620	0.0641	0.3334
rs2157926	87108436	A	T	0.9392	5090	-0.0544	0.0494	0.2708	0.2716	0.9880	0.0388	0.0406	0.3399
rs10233247	87109774	A	G	0.0238	0	NA	NA	NA	NA	NA	-0.0639	0.0653	0.3273
rs10275831	87113043	C	T	0.0238	0	NA	NA	NA	NA	NA	-0.0634	0.0654	0.3324
rs10246878	87113577	A	G	0.7735	0	NA	NA	NA	NA	NA	0.0193	0.0232	0.4057
rs10267099	87116696	A	G	0.2271	5382	-0.0195	0.0232	0.4013	0.4009	0.9994	-0.0194	0.0232	0.4015
rs10486996	87116897	A	G	0.0397	0	NA	NA	NA	NA	NA	-0.0050	0.0506	0.9220
rs12539395	87117792	C	T	0.0635	0	NA	NA	NA	NA	NA	-0.0282	0.0398	0.4794
rs11973812	87123053	C	G	0.0238	0	NA	NA	NA	NA	NA	-0.0632	0.0653	0.3336
rs7810499	87124382	C	T	0.9396	0	NA	NA	NA	NA	NA	-0.0081	0.0444	0.8543
rs2373575	87124417	A	G	0.0121	0	NA	NA	NA	NA	NA	0.2274	0.0939	0.0158
rs7790722	87131159	A	T	0.0635	0	NA	NA	NA	NA	NA	-0.0281	0.0399	0.4806
rs10952898	87134531	C	T	0.9763	0	NA	NA	NA	NA	NA	0.0615	0.0656	0.3483
rs2157930	87136804	A	G	0.7735	0	NA	NA	NA	NA	NA	0.0192	0.0232	0.4080
rs2157929	87140076	A	G	0.0635	0	NA	NA	NA	NA	NA	-0.0279	0.0399	0.4848
rs12540931	87140771	C	T	0.9365	0	NA	NA	NA	NA	NA	0.0280	0.0399	0.4835
rs6957599	87153817	A	G	0.9764	0	NA	NA	NA	NA	NA	0.0619	0.0657	0.3464

Association with SQRT transformed MDRD

SNP rs number	position on build 36	allele1 (on + strand of reference sequence)	allele2	frequency of allele 2	number of observed genotypes	PLINK analysis: effect of allele 2 (beta)	PLINK analysis: SE for beta	PLINK analysis: P value	Permutation tests: P value for single SNP	Permutation tests: P value for all SNPs in ABCB1	Analysis of imputed genotypes: effect of allele 2 (beta)	Analysis of imputed genotypes: SE for beta	Analysis of imputed genotypes: P value
rs7796247	87162322	A	G	0.9764	0	NA	NA	NA	NA	NA	0.0613	0.0655	0.3496
rs1015415	87168037	A	T	0.0399	0	NA	NA	NA	NA	NA	-0.0063	0.0504	0.9005
rs6465118	87168359	A	G	0.9366	0	NA	NA	NA	NA	NA	0.0279	0.0399	0.4845
rs10278483	87169901	C	T	0.9770	0	NA	NA	NA	NA	NA	0.0572	0.0795	0.4706
rs2188530	87170374	A	G	0.0235	0	NA	NA	NA	NA	NA	-0.0606	0.0658	0.3569
rs17149864	87172320	A	G	0.0632	5374	-0.0252	0.0400	0.5291	0.5290	1.0000	-0.0262	0.0399	0.5119
rs2188528	87175569	A	T	0.0399	0	NA	NA	NA	NA	NA	-0.0061	0.0504	0.9044
rs10261685	87182354	A	C	0.0235	0	NA	NA	NA	NA	NA	-0.0585	0.0655	0.3717
rs6972098	87190285	C	T	0.9366	0	NA	NA	NA	NA	NA	0.0276	0.0399	0.4882
rs976602	87190884	C	T	0.9366	0	NA	NA	NA	NA	NA	0.0277	0.0399	0.4871
rs17329325	87191012	C	T	0.0128	0	NA	NA	NA	NA	NA	-0.1025	0.0951	0.2806
rs6945109	87194362	A	G	0.0120	0	NA	NA	NA	NA	NA	0.2263	0.0949	0.0176
rs10486997	87196082	A	G	0.9765	0	NA	NA	NA	NA	NA	0.0593	0.0656	0.3658
rs12537294	87196454	C	T	0.9765	0	NA	NA	NA	NA	NA	0.0609	0.0657	0.3536
rs2188523	87197441	A	G	0.9765	0	NA	NA	NA	NA	NA	0.0605	0.0657	0.3565

chr, chromosome
snp, rs number
phypos, position **on build 35**
allele1, allele2, the two alleles on the + strand of reference sequence
freq2, the frequency of allele2
NOBS, the number of observed genotypes at this SNP (0 means a completely imputed SNP)
ebeta, estimated (posterior mean) additive effect of allele2
sebeta, a posterior standard deviation for beta, which is a proxy for the standard error
pbeta, a (Bayesian proxy for the) two-tailed p-value
BETA, PLINK estimate of beta
SE, the PLINK standard error for BETA
P, PLINK p-value using classical asymptotics
EMP1, PLINK permutation p-value for that SNP
EMP2, PLINK permutation p-value that is correct for multiple testing

SNPs highlighted in bold are the ones that were measured in the Seychelles sample.
Note: in all cases beta is measured in units of phenotypic standard deviations, after transformation and correction.
